# Modified Del Nido Cardioplegia with a 1:4 Crystalloid-to-Blood Ratio Versus Blood-Based St. Thomas Cardioplegia in Isolated Aortic Valve Replacement

**DOI:** 10.3390/jcdd13060263

**Published:** 2026-06-11

**Authors:** Peter Jakub, Tomáš Toporcer, Matúš Marcin, Michal Trebišovský, Anton Bereš, Marián Homola, Štefan Lukačín, Adrián Kolesár

**Affiliations:** Department of Heart Surgery, East Slovak Institute for Cardiovascular Diseases and Medical Faculty, Pavol Jozef Šafárik University, 04011 Košice, Slovakia; peterjakub24@gmail.com (P.J.); mmarcin@vusch.sk (M.M.); mtrebisovsky@vusch.sk (M.T.); aberes@vusch.sk (A.B.); mhomola@vusch.sk (M.H.); slukacin@vusch.sk (Š.L.); akolesar@vusch.sk (A.K.)

**Keywords:** myocardial protection, modified Del Nido cardioplegia, blood-based St. Thomas cardioplegia, aortic valve replacement, myocardial injury markers

## Abstract

The aim of this study was to retrospectively compare modified Del Nido and blood-based St. Thomas cardioplegia in adult patients undergoing isolated aortic valve replacement (AVR). This retrospective study included adult patients undergoing isolated AVR because of aortic valve stenosis between 2024 and 2025. Patients were stratified into blood-based St. Thomas and modified Del Nido groups. The main modification of the Del Nido solution was the adjustment of the crystalloid-to-blood ratio to 1:4. Preoperative and perioperative variables, as well as postoperative biomarkers, including high-sensitivity troponin I, creatine kinase (CK), CK-MB, and lactate, were analyzed. A total of 93 patients were included in the study (blood-based St. Thomas: *n* = 22; modified Del Nido: *n* = 71). No significant differences were observed in cardiopulmonary bypass time [98 min (IQR 84–110) vs. 90 min (IQR 74–110); *p* = 0.184] or aortic cross-clamp time [75 min (IQR 67–86) vs. 73 min (IQR 62–87); *p* = 0.345]. High-sensitivity troponin I levels at 24 h were numerically, but not statistically significantly, lower in the blood-based St. Thomas group [1961 ng/L (IQR 1367–4423) vs. 2819 ng/L (IQR 1698–5054); *p* = 0.240]. CK levels at 6 h were comparable between the groups [8.4 μkat/L (IQR 6.5–10.1) vs. 8.5 μkat/L (IQR 6.0–12.7); *p* = 0.632], as were CK-MB and lactate levels at all evaluated time points. In exploratory multivariable analyses adjusted for age, sex, preoperative LVEF, cardiopulmonary bypass time, and aortic cross-clamp time, cardioplegia type was not independently associated with postoperative biomarker levels. The less frequent dosing and membrane-stabilizing properties of modified Del Nido cardioplegia did not translate into statistically significant clinical or biochemical advantages in the setting of relatively short, isolated AVR procedures.

## 1. Introduction

The term cardioplegia (from cardio, heart, and plegia, paralysis) was first introduced by Lam in 1957; however, the concept of induced cardiac arrest originates from the earlier experimental work of the British physiologist Sidney Ringer [1,2]. Currently, between 61.6 and 123.2 per 100,000 individuals undergo cardiac surgery annually, based on World Bank World Development Indicators, the majority of whom undergo procedures performed under cardiac arrest induced by the administration of a cardioplegic solution [3]. The principal objectives of hypothermic cardioplegia are to achieve immediate and sustained electromechanical quiescence, to ensure rapid and uniform myocardial cooling, to reduce myocardial oxygen demand, and to facilitate the periodic washout of metabolic inhibitors [4]. Early cardioplegic solutions primarily contained potassium citrate, which induced prolonged depolarization of the myocardium, leading to calcium sequestration, reduced contractility, and diastolic arrest [5]. Currently, the most commonly used cardioplegic solution in adult patients is St. Thomas cardioplegia, which contains sodium, potassium, chloride, and bicarbonate [5,6]. The use of conventional potassium-based cardioplegia leads to spontaneous sodium influx, resulting in progressive membrane depolarization and subsequent accumulation of intracellular calcium ions. The ensuing release of additional calcium from the sarcoplasmic reticulum causes calcium overload, ultimately contributing to ischemia–reperfusion injury [7]. Subsequently, Del Nido cardioplegia was developed in the 1980s and 1990s for use in the immature myocardium and contains mannitol for free radical scavenging, magnesium sulfate for calcium channel blockade, and lidocaine as an antiarrhythmic component [4,7,8]. Del Nido cardioplegia was originally developed for pediatric patients; however, its use has increasingly been extended to adult populations across multiple centers [2,5,7,9,10,11,12,13,14]. Interestingly, despite its widespread clinical use, the effects of Del Nido cardioplegia on cellular processes have not yet been fully elucidated experimentally [8]. Some studies, however, report variability in the effectiveness of different cardioplegic solutions for myocardial protection across diverse patient populations. These differences have been attributed to variations in anatomical characteristics and the incidence of comorbidities associated with interpopulation differences [7].

The aim of this study was to retrospectively compare the safety and effectiveness of modified Del Nido cardioplegia with a 1:4 crystalloid-to-blood ratio and blood-based St. Thomas cardioplegia in adult patients undergoing isolated aortic valve replacement because of aortic valve stenosis.

## 2. Material and Methods

### 2.1. Inclusion Characteristics and Endpoints

All patients who underwent aortic valve replacement (AVR) for severe aortic stenosis at our institution between January 2024 and December 2025 were retrospectively included in this study. Patients undergoing concomitant surgical procedures, as well as those undergoing reoperation, were excluded. Preoperative high-sensitivity troponin (hs-TnI) levels were obtained from medical records. Patients with unavailable preoperative hs-TnI values or with hs-TnI levels >100 ng/L were excluded.

The following perioperative data were collected: type of cardioplegic solution used, age at the time of surgery, sex, preoperative creatinine level, body surface area (BSA), body mass index (BMI), presence of diabetes mellitus, hypertension, renal dysfunction, coronary artery disease, pulmonary hypertension, New York Heart Association (NYHA) functional class, EuroSCORE II, prosthesis type (biological vs. mechanical), surgical approach (median sternotomy (MS), partial upper mini-sternotomy (PUMS), or right anterior thoracotomy (RAT)), cardiopulmonary bypass (CPB) time, and aortic cross-clamp time. Left ventricular ejection fraction (LVEF) was assessed preoperatively and 24 h postoperatively, and the perioperative change in LVEF was calculated. In addition, ventricular fibrillation during CPB weaning was recorded. The time to spontaneous rhythm restoration was assessed only in patients who developed a spontaneous cardiac rhythm after aortic cross-clamp removal without the need for defibrillation and without the requirement for pacing. In these patients, the interval from cross-clamp removal to the onset of spontaneous rhythm was documented. The presence of spontaneous cardiac activity after cross-clamp removal without the need for pacing was also recorded. Serum levels of high-sensitivity troponin I, creatine kinase (CK), creatine kinase-myocardial band (CK-MB), and lactate were obtained at four time points: preoperatively and at 6, 24, and 48 h postoperatively.

### 2.2. Myocardial Protection

The choice of cardioplegic solution was left entirely to the discretion of the operating surgeon and was not guided by strict predefined criteria. Blood-based St. Thomas cardioplegia was prepared by aseptically withdrawing 50 mL of St. Thomas solution (Solutio Thomas cum procaino Ardeapharma; Ardeapharma a.s., Ševětín, Czech Republic) (containing 162.65 g/L magnesium chloride hexahydrate, 59.6 g/L potassium chloride, and 13.6 g/L procaine hydrochloride; pH 3.2–4.5; osmotic pressure 6200 kPa; with sodium metabisulfite as an excipient with a known effect), which was subsequently added to 450 mL of Ringer’s solution (Ringer’s Braun Injection; B. Braun Melsungen AG, Melsungen, Germany) pre-cooled to 4 °C and thoroughly mixed. The pH of the resulting solution was then adjusted to 7.8 by the aseptic addition of sodium bicarbonate. The solution was maintained at 3–4 °C and mixed with cold blood in a 1:4 ratio (crystalloid: blood). The final blood cardioplegia was administered as an initial dose of 15–20 mL/kg, followed by repeated doses every 20 min in volumes of 150–200 mL.

Modified Del Nido cardioplegia was prepared by mixing 1000 mL of Plasma-Lyte (Baxter Holding B.V., Utrecht, The Netherlands) with the following additives: 60 mL of 7.45% KCl (Kaliumchlorid B. Braun 7.45%; B. Braun Melsungen AG, Melsungen, Germany, 26 mL of 4.2% NaHCO_3_ (Hydrogenuhličitan sodný B. Braun 4.2%; B. Braun Melsungen AG, Melsungen, Germany), 20 mL of 10% MgSO_4_ (Magnesium sulfate Kalceks 100 mg/mL; AS KALCEKS, Riga, Latvia), 16 mL of 20% mannitol (Mannitol Fresenius Kabi 20%; Fresenius Kabi s.r.o., Prague, Czech Republic), and 6.5 mL of 2% lidocaine (Lidocain EGIS 20 mg/mL; Egis Pharmaceuticals PLC, Budapest, Hungary). The solution was cooled to 3–4 °C and mixed with cold blood in a 1:4 ratio (crystalloid: blood). The final blood cardioplegia was administered as an initial dose of 1800 mL, followed by repeated doses every 90 min in volumes of 600 mL.

### 2.3. Statistical Analysis

Patients were divided into two groups according to the cardioplegic solution used: blood-based St. Thomas cardioplegia and modified Del Nido cardioplegia. Because of the relatively small sample size, particularly in the St. Thomas group, and the expected non-normal distribution of several continuous variables, continuous data are presented as medians and interquartile ranges (IQRs). Categorical variables are presented as percentages, calculated as the number of positive cases divided by the number of patients for whom the respective parameter was available, with absolute numbers shown in parentheses. Between-group comparisons of continuous variables were performed using the Mann–Whitney U test. Categorical variables were analyzed using the chi-square test or Fisher’s exact test, as appropriate. For categorical variables with more than two categories, the Fisher–Freeman–Halton exact test was used when expected cell counts were low. Changes in high-sensitivity troponin I (hs-TnI), creatine kinase (CK), CK-MB, and lactate levels over four time points were analyzed using repeated-measures ANOVA. The assumption of sphericity was assessed using Mauchly’s test. When sphericity was violated, Greenhouse–Geisser correction was applied. Pairwise comparisons between time points were performed with Bonferroni adjustment.

In addition, exploratory multivariable linear regression analyses were performed to evaluate the association between cardioplegia type and postoperative biomarker levels after adjustment for potential confounders. The adjusted covariates included age, sex, preoperative left ventricular ejection fraction, cardiopulmonary bypass time, and aortic cross-clamp time. Because biomarker levels showed a non-normal distribution, logarithmically transformed values of high-sensitivity troponin I, creatine kinase, CK-MB, and lactate were used as dependent variables. For these exploratory adjusted analyses, selected postoperative time points were analyzed rather than all repeated measurements in order to limit the number of regression models relative to the modest sample size and to reduce the risk of overfitting. The selected time points were chosen to reflect the clinically most relevant postoperative biomarker elevations. These analyses were considered exploratory because of the limited sample size and the unequal distribution of patients between the study groups. Statistical analysis was performed using Microsoft Excel for Microsoft 365 (Microsoft Corporation, Redmond, WA, USA) and IBM SPSS Statistics version 20 (IBM Corp., Armonk, NY, USA). A *p*-value < 0.05 was considered statistically significant.

## 3. Results

### 3.1. Baseline and Perioperative Characteristics

**Figure 1:**
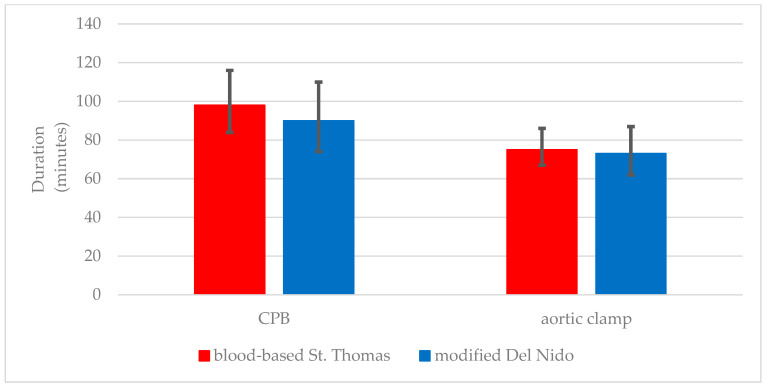
Comparison of cardiopulmonary bypass time and aortic cross-clamp time in patients receiving blood-based St. Thomas and modified Del Nido cardioplegia (CPB—cardio-pulmonary bypass).

A total of 93 patients were included in the study. Blood-based St. Thomas cardioplegia was used in 22 patients, whereas modified Del Nido cardioplegia was used in 71 patients. The two groups did not differ significantly in baseline demographic and clinical characteristics. Age was comparable between the St. Thomas and Del Nido groups [66 years (IQR 58–72) vs. 67 years (IQR 62–72), *p* = 0.423], as were female sex [45% (10/22) vs. 48% (34/71), *p* = 0.842], body surface area [2.0 m^2^ (IQR 1.9–2.1) vs. 2.0 m^2^ (IQR 1.8–2.1), *p* = 0.539], body mass index [32 kg/m^2^ (IQR 28–35) vs. 29 kg/m^2^ (IQR 27–33), *p* = 0.313], and creatinine levels [78 μmol/L (IQR 61–90) vs. 76 μmol/L (IQR 68–92), *p* = 0.638]. The prevalence of comorbidities was also similar between the groups, including diabetes mellitus [18% (4/22) vs. 25% (18/71), *p* = 0.489], hypertension [86% (19/22) vs. 92% (65/71), *p* = 0.472], renal dysfunction [32% (7/22) vs. 41% (29/71), *p* = 0.448], coronary artery disease [50% (11/22) vs. 52% (37/71), *p* = 0.862], and pulmonary hypertension [27% (6/22) vs. 26% (18/70), *p* = 0.885]. NYHA functional class was comparable between the groups [2 (IQR 2–3) vs. 2 (IQR 2–3), *p* = 0.710], as was EuroSCORE II [2.1% (IQR 1.1–3.5) vs. 2.0% (IQR 1.3–2.8), *p* = 0.975]. There were no significant differences in procedural characteristics. The proportion of patients receiving a biological prosthesis was 82% (18/22) in the blood-based St. Thomas group and 92% (65/71) in the modified Del Nido group (*p* = 0.198). The distribution of surgical approaches, including median sternotomy, partial upper mini-sternotomy and right anterior thoracotomy, was similar between groups [59%/36%/5% (13/8/1) vs. 44%/55%/1% (31/39/1), *p* = 0.648]. Cardiopulmonary bypass duration was 98 min (IQR 84–110) in the blood-based St. Thomas group and 90 min (IQR 74–110) in the modified Del Nido group (*p* = 0.184), while aortic cross-clamp time was 75 min (IQR 67–86) and 73 min (IQR 62–87), respectively (*p* = 0.345) (Table 1, Figure 1).

### 3.2. Temporal Changes in Postoperative Biomarkers

Repeated-measures ANOVA demonstrated a significant change in hs-TnI levels over time. Median hs-TnI increased from 9.0 (IQR 6.5–15.4) before surgery to 3658 (IQR 2178–6926) at 6 h, followed by a decrease to 2656 (IQR 1672–4587) at 24 h and 1641 (IQR 926–3820) at 48 h. Mauchly’s test indicated a violation of the assumption of sphericity, W = 0.286, *p* < 0.001; therefore, the Greenhouse–Geisser correction was applied. The time effect remained statistically significant, F (1.763, 162.187) = 76.457, *p* < 0.001, partial η^2^ = 0.454. Bonferroni-adjusted pairwise comparisons showed significant differences between all time points, with peak hs-TnI values observed at 6 h after surgery.

Repeated-measures ANOVA demonstrated a statistically significant change in CK levels over time. Median CK increased from 1.6 (IQR 1.1–2.4) before surgery to 8.5 (IQR 6.1–12.0) at 6 h and 9.2 (IQR 6.6–12.7) at 24 h, followed by a decrease to 7.7 (IQR 5.0–11.4) at 48 h. Mauchly’s test showed violation of the assumption of sphericity, W = 0.205, *p* < 0.001; therefore, Greenhouse–Geisser correction was applied. The effect of time remained statistically significant, F (1.957, 180.018) = 66.378, *p* < 0.001, partial η^2^ = 0.419. Bonferroni-adjusted pairwise comparisons showed significantly higher CK levels at all postoperative time points compared with preoperative values (all *p* < 0.001). CK levels did not differ significantly between 6 and 24 h (*p* = 1.000) or between 6 and 48 h (*p* = 0.254), whereas values at 48 h were significantly lower than those at 24 h (*p* < 0.001).

Repeated-measures ANOVA was used to assess changes in CK-MB levels over four time points. Median CK-MB increased from 1.3 (IQR 0.9–2.1) before surgery to 25.4 (IQR 17.1–38.9) at 6 h and 21.9 (IQR 15.2–32.9) at 24 h, followed by a decrease to 10.1 (IQR 6.9–16.3) at 48 h. Mauchly’s test demonstrated violation of the assumption of sphericity, W < 0.001, *p* < 0.001; therefore, Greenhouse–Geisser correction was applied. After Greenhouse–Geisser correction, the overall effect of time did not reach statistical significance, F (1.010, 92.958) = 3.656, *p* = 0.059, partial η^2^ = 0.038. Bonferroni-adjusted pairwise comparisons showed significantly higher CK-MB values at 6 h and 48 h compared with preoperative levels, and significantly lower values at 48 h compared with 6 h; however, given the non-significant overall Greenhouse–Geisser-corrected time effect, these pairwise findings should be interpreted cautiously.

Repeated-measures ANOVA demonstrated a statistically significant change in lactate levels over time. Median lactate increased from 0.9 (IQR 0.7–1.1) before surgery to 1.9 (IQR 1.5–2.9) at 6 h, followed by a decrease to 1.6 (IQR 1.2–2.6) at 24 h and 1.2 (IQR 0.9–1.5) at 48 h. Mauchly’s test showed violation of the assumption of sphericity, W = 0.530, *p* < 0.001; therefore, Greenhouse–Geisser correction was applied. The effect of time remained statistically significant, F (2.168, 199.419) = 50.099, *p* < 0.001, partial η^2^ = 0.353. Bonferroni-adjusted pairwise comparisons demonstrated significant differences between all time points, with lactate peaking at 6 h after surgery and subsequently decreasing at 24 and 48 h.

### 3.3. Comparison of Cardioplegia-Related Postoperative Outcomes

When evaluating potential consequences of cardioplegia type, ventricular fibrillation during cardiopulmonary bypass weaning was observed in 0% (0/7) of patients in the blood-based St. Thomas group and in 33% (10/30) of patients in the modified Del Nido group, without a statistically significant difference between the groups (*p* = 0.155). Time to spontaneous rhythm recovery was 4 min (IQR 3–4) in the blood-based St. Thomas group and 1 min (IQR 1–2) in the modified Del Nido group (*p* = 0.118). Spontaneous cardiac activity was recorded in 100% (4/4) and 78% (21/27) of patients with blood-based St. Thomas and modified Del Nido cardioplegia, respectively (*p* = 0.561). However, these parameters were available only in a limited subset of patients; therefore, both the statistical power and the interpretative value of these comparisons are substantially limited (Table 1).

**Figure 2:**
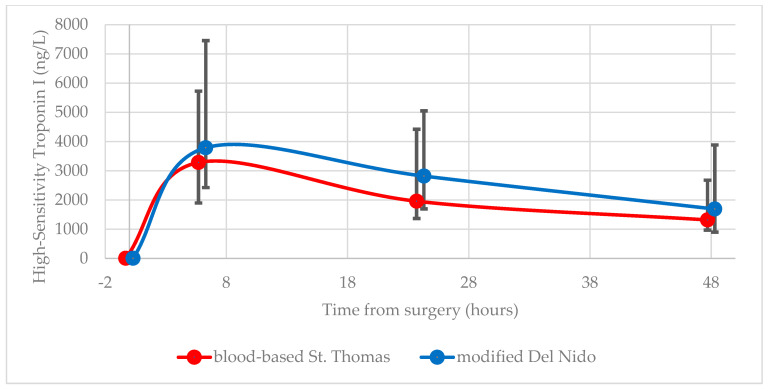
Postoperative dynamics of high-sensitivity troponin levels in patients receiving blood-based St. Thomas and modified Del Nido cardioplegia.

**Figure 3:**
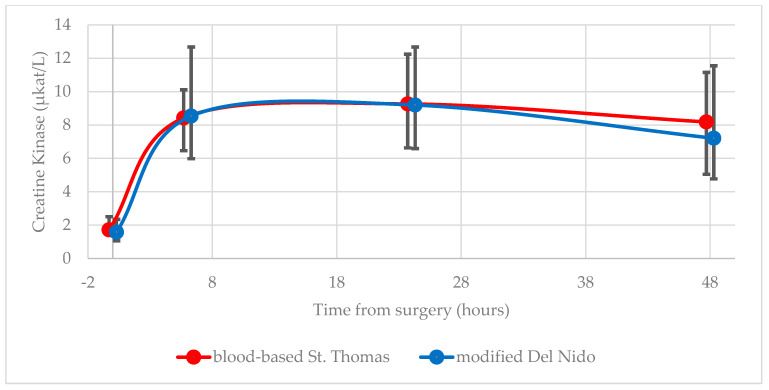
Postoperative dynamics of creatine kinase (CK) levels in patients receiving blood-based St. Thomas and modified Del Nido cardioplegia.

**Figure 4:**
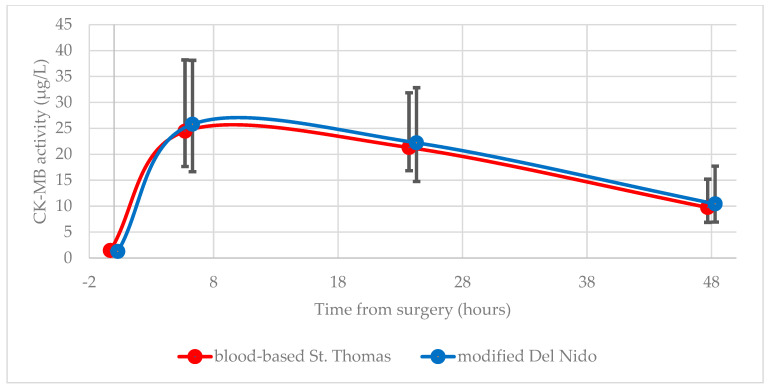
Postoperative dynamics of CK-MB levels in patients receiving blood-based St. Thomas and modified Del Nido cardioplegia (CK-MB—creatine kinase-myocardial band).

**Figure 5:**
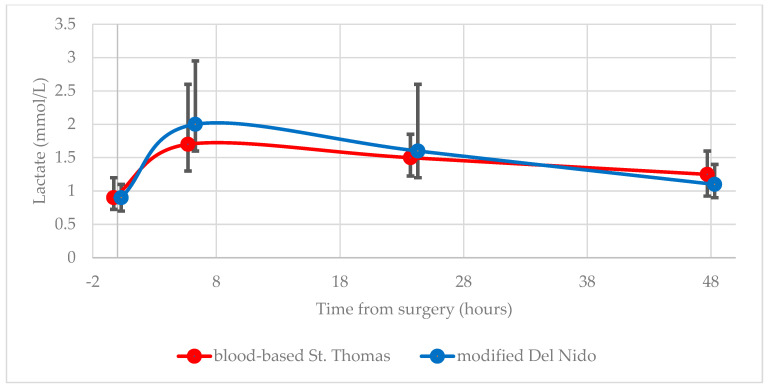
Postoperative dynamics of lactate levels in patients receiving blood-based St. Thomas and modified Del Nido cardioplegia.

No statistically significant differences were observed in postoperative cardiac biomarkers or lactate levels between the groups. High-sensitivity troponin I levels were comparable between the blood-based St. Thomas and modified Del Nido groups before surgery [8.8 ng/L (IQR 6.3–11.1) vs. 9.3 ng/L (IQR 6.6–16.1), *p* = 0.458], at 6 h [3287 ng/L (IQR 1897–5727) vs. 3789 ng/L (IQR 2427–7452), *p* = 0.645], at 24 h [1961 ng/L (IQR 1367–4423) vs. 2819 ng/L (IQR 1698–5054), *p* = 0.240], and at 48 h after surgery [1319 ng/L (IQR 974–2679) vs. 1695 ng/L (IQR 906–3885), *p* = 0.376] (Table 1, Figure 2). Similarly, CK levels did not differ significantly between the blood-based St. Thomas and modified Del Nido groups before surgery [1.7 μkat/L (IQR 1.4–2.5) vs. 1.6 μkat/L (IQR 1.1–2.4), *p* = 0.437], at 6 h [8.4 μkat/L (IQR 6.5–10.1) vs. 8.5 μkat/L (IQR 6.0–12.7), *p* = 0.632], at 24 h [9.3 μkat/L (IQR 6.6–12.2) vs. 9.2 μkat/L (IQR 6.6–12.7), *p* = 0.993], or at 48 h after surgery [8.2 μkat/L (IQR 5.1–11.2) vs. 7.2 μkat/L (IQR 4.8–11.6), *p* = 0.655] (Table 1, Figure 3). CK-MB activity was also comparable between the groups at all time points: before surgery [1.5 μg/L (IQR 0.9–2.2) vs. 1.3 μg/L (IQR 0.9–2.2), *p* = 0.860], at 6 h [25.5 μg/L (IQR 17.7–38.2) vs. 25.8 μg/L (IQR 16.6–38.1), *p* = 0.899], at 24 h [21.3 μg/L (IQR 16.8–31.9) vs. 22.2 μg/L (IQR 14.8–32.8), *p* = 0.821], and at 48 h after surgery [9.8 μg/L (IQR 6.9–15.2) vs. 10.4 μg/L (IQR 6.9–17.7), *p* = 0.671] (Table 1, Figure 4). No significant differences were observed in lactate levels between the blood-based St. Thomas and modified Del Nido groups before surgery [0.9 mmol/L (IQR 0.7–1.2) vs. 0.9 mmol/L (IQR 0.7–1.1), *p* = 0.699], at 6 h [1.7 mmol/L (IQR 1.3–2.6) vs. 2.0 mmol/L (IQR 1.6–3.0), *p* = 0.102], at 24 h [1.5 mmol/L (IQR 1.2–1.9) vs. 1.6 mmol/L (IQR 1.2–1.6), *p* = 0.382], or at 48 h after surgery [1.3 mmol/L (IQR 0.9–1.6) vs. 1.1 mmol/L (IQR 0.9–1.4), *p* = 0.540] (Table 1, Figure 5).

### 3.4. Multivariable Linear Regression

**Figure 6:**
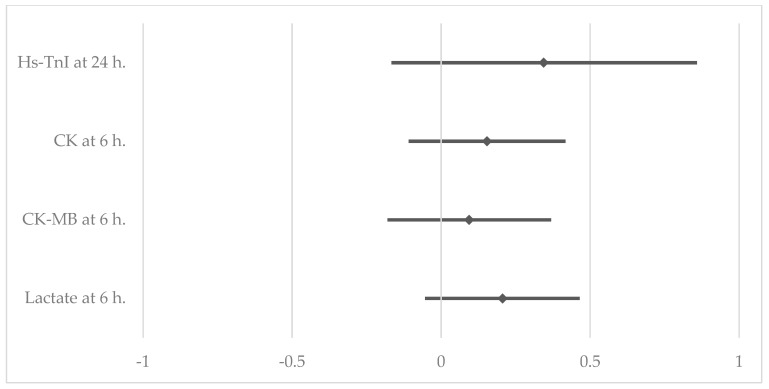
Exploratory multivariable linear regression analysis of the association between the use of modified Del Nido cardioplegia, compared with blood-based St. Thomas cardioplegia, and selected postoperative biomarker levels. Points represent regression coefficients (B), and horizontal lines represent 95% confidence intervals. Biomarker values were logarithmically transformed. Models were adjusted for age, sex, preoperative LVEF, CPB time, and aortic cross-clamp time (hs-TnI—high-sensitivity troponin I; CK—creatine kinase; CK-MB—creatine kinase-myocardial band; LVEF—left ventricular ejection fraction; CPB—cardiopulmonary bypass).

Exploratory multivariable linear regression analyses were performed to assess the association between cardioplegia type and selected postoperative biomarker levels after adjustment for age, sex, preoperative LVEF, CPB time, and aortic cross-clamp time. Cardioplegia type was not independently associated with log-transformed hs-TnI levels at 24 h after surgery (B = 0.344, 95% CI −0.167 to 0.856, *p* = 0.184). The overall model did not reach statistical significance (F (6,85) = 1.702, *p* = 0.130; adjusted R^2^ = 0.044) (Table 2, Figure 6).

Similarly, cardioplegia type was not independently associated with log-transformed CK levels at 6 h (B = 0.154, 95% CI −0.109 to 0.417, *p* = 0.248), CK-MB levels at 6 h (B = 0.094, 95% CI −0.180 to 0.369, *p* = 0.495), or lactate levels at 6 h after surgery (B = 0.206, 95% CI −0.054 to 0.465, *p* = 0.118). The overall models were statistically significant for CK (F (6,86) = 4.627, *p* < 0.001; adjusted R^2^ = 0.191) and CK-MB (F (6,86) = 4.601, *p* < 0.001; adjusted R^2^ = 0.190), but not for lactate (F (6,86) = 0.952, *p* = 0.463; adjusted R^2^ = −0.003). Among the included covariates, CPB time was independently associated with higher 6 h CK levels (B = 0.014, *p* = 0.022), while female sex (B = 0.315, *p* = 0.007) and CPB time (B = 0.019, *p* = 0.003) were independently associated with higher 6 h CK-MB levels (Table 2, Figure 6).

## 4. Discussion

### 4.1. Aortic Cross-Clamp Time and CPB Duration

A meta-analysis including 681 and 654 patients in the Del Nido cardioplegia and St. Thomas groups, respectively, demonstrated a significantly shorter aortic cross-clamp time with the use of Del Nido cardioplegia; however, this finding was not confirmed in pediatric patients [5]. Another earlier published meta-analysis similarly reported a reduction in cardiopulmonary bypass duration of approximately 15 min [11]. A smaller retrospective study including 200 patients also demonstrated a reduction in cardiopulmonary bypass duration with the use of Del Nido cardioplegia (137 ± 7 vs. 177 ± 7 min, *p* = 0.001) [10]. A reduction in cardiopulmonary bypass duration with the use of Del Nido cardioplegia compared with St. Thomas cardioplegia has also been documented in veterinary applications, specifically during mitral valve surgery in dogs [4]. On the other hand, a randomized study including patients undergoing CABG and valve surgery did not demonstrate a difference in cardiopulmonary bypass duration between Del Nido cardioplegia and blood-based St. Thomas cardioplegia (89 ± 27 min vs. 67 ± 24 min) [9]. Similarly, another randomized study including a broader spectrum of cardiac surgical procedures did not demonstrate a statistically significant difference in cardiopulmonary bypass duration between Del Nido cardioplegia and St. Thomas cardioplegia (85 ± 38 min vs. 87 ± 44 min, *p* = 0.631) [2]. In the present study, the surgical procedure was highly uniform across the entire cohort; nevertheless, despite a numerically shorter median cardiopulmonary bypass duration in the modified Del Nido cardioplegia group, the difference between the groups did not reach statistical significance.

Multiple meta-analyses have demonstrated that, compared with St. Thomas cardioplegia, Del Nido cardioplegia is associated with a shorter cardiopulmonary bypass duration in both adult and pediatric patients [5,11]. The aforementioned smaller retrospective study, consistent with the findings of the meta-analysis, also reported a shorter aortic cross-clamp time with the use of Del Nido cardioplegia, decreasing from 105 ± 4 min with St. Thomas cardioplegia to 90 ± 4 min (*p* = 0.006) [10]. Compared with St. Thomas cardioplegia, Del Nido cardioplegia is associated with a shorter aortic cross-clamp time even in veterinary applications [4]. In two randomized studies, one including 40 patients in each group and another including a total of 133 patients, no difference in aortic cross-clamp time during cardiac surgery was observed between Del Nido cardioplegia and blood-based St. Thomas cardioplegia; in the former study, cross-clamp times were 74 ± 23 vs. 65 ± 24 min (*p* = 0.84), while in the latter study, they were 64 ± 25 vs. 67 ± 29 min (*p* = 0.582) [2,9]. Similarly, another smaller study including 100 adult and 120 pediatric patients did not demonstrate a difference in aortic cross-clamp time based on the type of cardioplegia used in either cohort [12]. Similarly, no differences were identified in postoperative hemoglobin, urea, or creatinine levels [9]. As observed for cardiopulmonary bypass duration, the 2 min reduction in aortic cross-clamp time associated with single-dose modified Del Nido cardioplegia did not reach statistical significance between the groups.

### 4.2. Postoperative Dynamics of Cardiac-Specific Biomarkers

A study employing propensity score matching in patients with impaired ejection fraction undergoing valvular or combined cardiac procedures represents one of the few analyses demonstrating a significant benefit of Del Nido cardioplegia compared with cold blood cardioplegia. The authors reported significantly lower postoperative troponin elevation in the Del Nido group both at 12 h (median (IQR): 523.2 (349.1–740.4) pg/mL vs. 787.6 (443.6–1689.0) pg/mL; *p* = 0.016) and at 36 h (median (IQR): 426.1 (337.2–492.1) pg/mL vs. 653.7 (398.8–1737.5) pg/mL; *p* = 0.044) after surgery [15]. A randomized study including 80 patients (40 receiving Del Nido cardioplegia and 40 receiving St. Thomas cardioplegia) demonstrated significantly lower CK-MB levels 5–6 h postoperatively in the Del Nido group (31 ± 23 IU vs. 51 ± 50 IU) [9]. A study focused on mitral valve replacement similarly demonstrated non-significantly lower troponin levels at 6, 24, and 48 h postoperatively with the use of Del Nido cardioplegia compared with St. Thomas cardioplegia [13]. Similarly, another randomized study did not demonstrate a statistically significant difference in troponin or CK-MB levels 24 h after surgery. The authors reported slightly higher cTnI and CK-MB levels in the St. Thomas cardioplegia group (5.02 ± 2.14 vs. 4.88 ± 2.66 ng/mL, *p* = 0.189; 28 ± 9 vs. 26 ± 11 ng/mL, *p* = 0.341), which is consistent with our findings [2]. The same study demonstrated significantly higher early postoperative levels of malondialdehyde in the St. Thomas cardioplegia group (4.85 ± 0.73 vs. 4.58 ± 0.82 nmol/mL, *p* = 0.037) and superoxide dismutase in the Del Nido cardioplegia group (110 ± 14 vs. 98 ± 15 U/mL, *p* = 0.027). However, malondialdehyde showed no statistically significant differences 24 h after surgery [2]. A smaller retrospective study focused on postoperative troponin levels demonstrated differences at both 24 and 48 h after surgery. In both time intervals, lower troponin levels were observed with the use of Del Nido cardioplegia (1.44 ± 0.98 vs. 2.54 ± 2.37 ng/mL and 0.84 ± 0.61 vs. 1.42 ± 1.59 ng/mL at 24 and 48 h postoperatively, respectively) [10]. In our study, lower elevations in all three assessed cardiac-specific biomarkers (troponin, CK, and CK-MB) were observed with the use of blood-based St. Thomas cardioplegia. However, this non-significant difference was evident only during the first 24 h postoperatively, whereas at 48 h after surgery, the levels of these biomarkers were nearly identical in both compared groups.

### 4.3. Spontaneous Rhythm Recovery and Defibrillation Requirement

Two independent meta-analyses have demonstrated a lower requirement for defibrillation with the use of Del Nido cardioplegia compared with the St. Thomas cardioplegia in both adult and pediatric patients [5,11]. A randomized study including 40 patients in each group demonstrated a higher rate of spontaneous sinus rhythm restoration (95% vs. 73%, *p* = 0.05) and a lower requirement for defibrillation (5% vs. 18%, *p* < 0.001) with Del Nido cardioplegia compared with St. Thomas cardioplegia [9]. Another smaller study demonstrated a lower risk of defibrillation requirement in adult patients, a finding that was not observed in the pediatric population [12]. Another randomized study did not demonstrate a statistically significant difference in postoperative arrhythmogenic complications. The authors reported ventricular arrhythmias following aortic cross-clamp removal in 28% and 29% of patients, a requirement for defibrillation in 15% and 18%, and a need for pacing in 14% and 1% of patients for Del Nido cardioplegia and St. Thomas cardioplegia, respectively [2]. Sazzad et al. reported restoration of sinus rhythm within 2–4 min following the administration of Del Nido cardioplegia, which is consistent with our findings of 1.9 ± 1.34 min [7]. A retrospective study including 200 patients demonstrated a lower risk of requiring intraoperative cardioversion with the use of Del Nido cardioplegia compared with St. Thomas cardioplegia (14% vs. 33%, *p* = 0.001) [10]. In the present study, parameters related to rhythm recovery after cross-clamp removal were available only in a small subset of patients. Therefore, although numerical differences were observed between the groups, including a shorter time to spontaneous rhythm restoration with modified Del Nido cardioplegia, these findings should be considered exploratory and cannot be used to draw reliable conclusions regarding the effect of cardioplegia type on rhythm recovery.

While a retrospective propensity score-matched study reported a higher incidence of postoperative atrial fibrillation in the Del Nido cardioplegia group compared with St. Thomas cardioplegia (27% vs. 17%, *p* = 0.003), a prospective randomized study demonstrated a comparable incidence of this complication (9% vs. 12%) [2,7].

### 4.4. Impact on Left Ventricular Ejection Fraction and Survival

In the literature, propensity score-matched analyses in patients with preoperatively reduced LVEF have demonstrated a significantly lower incidence of a postoperative decline in LVEF greater than 5% with the use of Del Nido cardioplegia compared with cold blood cardioplegia (7% vs. 16%; *p* = 0.046) [15]. Another smaller study demonstrated higher postoperative left ventricular ejection fraction following mitral valve replacement with the use of Del Nido cardioplegia compared with St. Thomas cardioplegia (53 ± 7% vs. 45 ± 11%, *p* = 0.023) [13]. A randomized study including 133 patients did not demonstrate differences in early postoperative left ventricular ejection fraction (54 ± 7% vs. 52 ± 6%, *p* = 0.652) or in LVEF at 24 h after surgery (57 ± 9% vs. 56 ± 6% for Del Nido cardioplegia and St. Thomas cardioplegia, respectively; *p* = 0.684) when comparing the two cardioplegic strategies [2]. A study evaluating the need for intraoperative inotropic support and intra-aortic balloon counterpulsation following weaning from cardiopulmonary bypass did not demonstrate a difference between Del Nido cardioplegia and St. Thomas cardioplegia [10]. The same study also did not demonstrate an association between postoperative left ventricular ejection fraction and the type of cardioplegia used [10]. Our study also did not demonstrate that the choice of cardioplegia influenced changes in left ventricular ejection fraction.

A meta-analysis did not demonstrate a significant impact of cardioplegia type on patient survival (445 patients in the Del Nido cardioplegia cohort and 457 patients in the St. Thomas cohort) or on ICU length of stay (581 patients in the Del Nido cohort and 553 patients in the St. Thomas cohort). However, a shorter overall hospital stay was observed with the use of Del Nido cardioplegia (a total of 1011 patients included). Notably, with regard to hospital length of stay, adult patients appeared to benefit from St. Thomas cardioplegia, whereas pediatric patients showed more favorable outcomes with Del Nido cardioplegia [5]. Similarly, a propensity score-matched study including 307 matched pairs did not demonstrate differences in 30-day mortality or major adverse cardiac events (MACE), despite the Del Nido cardioplegia group comprising fewer emergent patients and a higher proportion of non-coronary artery bypass graft cases [7]. Another randomized study focusing on the duration of mechanical ventilation and Intensive Care Unit (ICU) stay reported both shorter ventilation time (166 ± 48 vs. 204 ± 77 min, *p* = 0.03) and reduced ICU length of stay (5.2 ± 0.6 vs. 6.05 ± 1.6 days, *p* = 0.003) with the use of Del Nido cardioplegia [9].

### 4.5. Cardioplegia Preparation

The composition of blood-based St. Thomas cardioplegia may vary slightly between institutions according to the literature. While the concentration of potassium chloride in the final crystalloid solution is relatively consistent, typically ranging from 80.5 to 90.5 mmol/L (79.95 mmol/L in our protocol), the concentration of magnesium chloride shows greater variability, ranging from 36.55 to 90.5 mmol/L (80 mmol/L in our setting) [2,9,10]. Some authors do not report the use of procaine; however, when included, it is typically administered at concentrations between 5.13 and 5.66 mmol/L, which is comparable to the 4.99 mmol/L used in our protocol [2,9,10]. The final dilution for the preparation of blood cardioplegia is consistently described by multiple authors as a 1:4 ratio, which is also employed at our institution. Most authors do not report pH standardization as applied in our protocol [2,9,10].

A key component of Del Nido cardioplegia is lidocaine, which, due to its prolonged duration of action, contributes to the stabilization of cellular membranes. This provides the added advantage of orchestrating a slower resumption of electrical activity after reperfusion, thereby allowing recovery of mitochondrial function and adenosine triphosphate (ATP) stores before intracellular Ca^2+^ pumps resume full activity. Magnesium further exerts a protective effect by competitively inhibiting Ca^2+^ influx into the cell [16]. On the other hand, a notable limitation is the heterogeneity of solution compositions reported in the literature.

Najmuddin et al. defined an optimal potassium concentration in the final blood-mixed solution at 24 mmol/L [17]. However, reported concentrations vary widely, ranging from 8.5 to 38.64 mmol/L, reflecting differences in the crystalloid composition (16–48 mmol/L) as well as in the crystalloid-to-blood mixing ratios [10,18,19,20]. Most commonly, the literature describes the use of 13 mL of potassium chloride added to 1000 mL of Plasma-Lyte, subsequently mixed with blood in a 4:1 ratio (crystalloid: blood), corresponding to a final potassium concentration of approximately 26 mmol/L in the administered cardioplegia [9,16,21,22,23,24]. In our variation, although a higher potassium concentration is present in the crystalloid solution, the use of a 1:4 crystalloid-to-blood mixing ratio, together with an assumed physiological potassium concentration in blood, results in a final delivered concentration of approximately 13.5 mmol/L.

Lidocaine, a hallmark of Del Nido cardioplegia, is responsible for the stabilization of cellular membranes, leading to decreased myocardial excitability. It is most commonly administered at a dose of 130 mg (6.5 mL of a 2% solution) per 1000 mL of crystalloid, which, when diluted in a 4:1 ratio (crystalloid: blood), results in a final concentration of approximately 0.415 mmol/L [9,21,22,23,25]. However, the literature reports a wide range of lidocaine concentrations in the final blood-mixed cardioplegia, primarily due to varying crystalloid-to-blood dilution ratios, ranging from 0.09 to 0.55 mmol/L [10,18]. The cardioplegia used in our protocol contains approximately 0.09 mmol/L lidocaine. Similar variability is observed for the remaining components. Bicarbonate concentrations in the crystalloid solution range from 5.6 to 16 mmol/L and from 2.46 to 12.8 mmol/L in the final administered solution [9,10,18,25]. Most commonly, a concentration of 13 mmol/L in the crystalloid and approximately 10 mmol/L in the final solution is reported [16,17,19,21,22,23,25]. Magnesium sulfate concentrations are most frequently reported around 7.7 mmol/L in the crystalloid, with a broader range from 1.5 to 10.1 mmol/L [9,10,16,18,21,22,23,24,25]. Mannitol, which contributes to the reduction in myocardial edema, is reported in crystalloid concentrations ranging from 10.5 to 22 mmol/L, most commonly around 17 mmol/L [2,9,10,16,18,19,21,22,23,25]. In contrast, the approach to calcium is consistent across authors, with consensus that it should be absent from the Del Nido solution [2,9,10,16,18,19,20,21,22,23,24,25].

### 4.6. Limitations

Several limitations of this study should be acknowledged. First, this was a retrospective single-center analysis with a relatively small sample size. No a priori sample size calculation was performed because the cohort size was determined by the number of eligible patients undergoing isolated AVR during the study period. Consequently, the study may have been underpowered to detect small or moderate differences between the groups, and the absence of statistically significant differences should be interpreted with caution. Second, the study population was unevenly distributed between the cardioplegia groups, reflecting institutional practice and surgeon preference during the study period, which may have introduced selection bias. Third, the absence of randomization limits the ability to control for potential confounding factors. In addition, data on ventricular fibrillation during CPB weaning, time to spontaneous rhythm restoration, and spontaneous cardiac activity after cross-clamp removal were available only in a limited subset of patients. Therefore, these parameters should be considered exploratory and cannot be used to draw reliable conclusions regarding the effect of cardioplegia type on rhythm recovery. Furthermore, only short-term perioperative and biochemical outcomes were evaluated, while long-term clinical outcomes, including postoperative arrhythmias, ventricular function recovery, and survival, were not assessed. Finally, myocardial protection was primarily evaluated using postoperative biomarker dynamics rather than advanced imaging or direct functional assessment of myocardial injury.

## 5. Conclusions

The presented results suggest that the effectiveness of blood-based St. Thomas cardioplegia and modified Del Nido cardioplegia is largely comparable in the setting of isolated aortic valve replacement. The use of blood-based St. Thomas cardioplegia was associated with a non-significant trend toward lower postoperative elevations in cardiac-specific biomarkers, including troponin, CK, and CK-MB. In contrast, the use of modified Del Nido cardioplegia was associated with a modest, non-significant reduction in cardiopulmonary bypass and aortic cross-clamp times. In exploratory multivariable analyses adjusted for age, sex, preoperative LVEF, cardiopulmonary bypass time, and aortic cross-clamp time, cardioplegia type was not independently associated with postoperative biomarker levels.

The less frequent administration and membrane-stabilizing properties conferred by the optimized composition of modified Del Nido cardioplegia, compared with blood-based St. Thomas cardioplegia, do not appear to translate into statistically significant improvements in the evaluated parameters in the context of relatively short cardiac surgical procedures.

## Figures and Tables

**Table 1:** Statistical comparison of the studied parameters in patients receiving blood-based St. Thomas and modified Del Nido cardioplegia (BMI—body mass index; BSA—body surface area; CK-MB—creatine kinase-myocardial band; CPB—cardio-pulmonary bypass; LVEF—left ventricular ejection fraction; MS—medial sternotomy; NYHA—New York Heart Association functional classification; PUMS–partial upper mini-sternotomy; RAT–right anterior thoracotomy).

Parameter	Blood-Based St. Thomas	Modified Del Nido	*p*
Age	66 (IQR 58–72)	67 (IQR 62–72)	0.423
Sex (woman)	45% (10/22)	48% (34/71)	0.842
BSA (m^2^)	2 (IQR 1.9–2.1)	2 (IQR 1.8–2.1)	0.539
BMI (kg/m^2^)	32 (IQR 28–35)	29 (IQR 27–33)	0.313
Creatinine (μmol/L)	78 (IQR 61–90)	76 (IQR 68–92)	0.638
Diabetes mellitus	18% (4/22)	25% (18/71)	0.489
Hypertension	86% (19/22)	92% (65/71)	0.472
Renal dysfunction	32% (7/22)	41% (29/71)	0.448
Coronary artery disease	50% (11/22)	52% (37/71)	0.862
Pulmonary hypertension	27% (6/22)	26% (18/70)	0.885
NYHA	2 (IQR 2–3)	2 (IQR 2–3)	0.710
EuroSCORE II (%)	2.1 (IQR 1.1–3.5)	2 (IQR 1.3–2.8)	0.975
Prosthesis type (biological)	82% (18/22)	92% (65/71)	0.198
Surg. Approach (MS/PUMS/RAT)	59%/36%/5% (13/8/1)	44%/55%/1% (31/39/1)	0.648
CPB (min)	98 (IQR 84–110)	90 (IQR 74–110)	0.184
Aortic clamp (min)	75 (IQR 67–86)	73 (IQR 62–87)	0.345
ΔLVEF (%)	−4 (IQR −5 to −4)	−4 (IQR −4 to −1)	0.208
VF during CPB weaning	0% (0/7)	33% (10/30)	0.155
Time to spontaneous rhythm (min)	4 (IQR 3–4)	1 (IQR 1–2)	0.118
Spontaneous cardiac activity, *n* (%)	100% (4/4)	78% (21/27)	0.561
High-sensitivity Troponin I (ng/L)			
Before surgery	8.8 (IQR 6.3–11.1)	9.3 (IQR 6.6–16.1)	0.458
Six h after surgery	3287 (IQR 1897–5727)	3789 (IQR 2427–7452)	0.645
One day after surgery	1961 (IQR 1367–4423)	2819 (IQR 1698–5054)	0.240
Two days after surgery	1319 (IQR 974–2679)	1695 (IQR 906–3885)	0.376
Creatine kinase (μkat/L)			
Before surgery	1.7 (IQR 1.4–2.5)	1.6 (IQR 1.1–2.4)	0.437
Six h after surgery	8.4 (IQR 6.5–10.1)	8.5 (IQR 6–12.7)	0.632
One day after surgery	9.3 (IQR 6.6–12.2)	9.2 (IQR 6.6–12.7)	0.993
Two days after surgery	8.2 (IQR 5.1–11.2)	7.2 (IQR 4.8–11.6)	0.655
CK-MB activity (μg/L)			
Before surgery	1.5 (IQR 0.9–2.2)	1.3 (IQR 0.9–2.2)	0.860
Six h after surgery	25.5 (IQR 17.7–38.2)	25.8 (IQR 16.6–38.1)	0.899
One day after surgery	21.3 (IQR 16.8–31.9)	22.2 (IQR 14.8–32.8)	0.821
Two days after surgery	9.8 (IQR 6.9–15.2)	10.4 (IQR 6.9–17.7)	0.671
Lactate (mmol/L)			
Before surgery	0.9 (IQR 0.7–1.2)	0.9 (IQR 0.7–1.1)	0.699
Six h after surgery	1.7 (IQR 1.3–2.6)	2 (IQR 1.6–3)	0.102
One day after surgery	1.5 (IQR 1.2–1.9)	1.6 (IQR 1.2–1.6)	0.382
Two days after surgery	1.3 (IQR 0.9–1.6)	1.1 (IQR 0.9–1.4)	0.540

**Table 2:** Exploratory multivariable linear regression analysis of postoperative biomarker levels. (CK—creatine kinase; CK-MB—creatine kinase-myocardial band; CPB—cardiopulmonary bypass; hs-TnI—high-sensitivity troponin I; LVEF—left ventricular ejection fraction).

Parameter	*p*-Value	Regression Coefficient (B)	95%CI	
			Lower	Upper
Effect on log-transformed hs-TnI levels at 24 h after surgery				
Modified Del Nido cardioplegia	0.184	0.344	−0.167	0.856
Age	0.222	−0.017	−0.044	0.010
Female sex	0.354	−0.200	−0.626	0.227
Preoperative LVEF	0.133	−2.110	−4.876	0.656
CPB time	0.175	0.016	−0.007	0.039
Aortic cross-clamp time	0.516	−0.010	−0.040	0.020
Effect on log-transformed CK levels at 6 h after surgery				
Modified Del Nido cardioplegia	0.248	0.154	−0.109	0.417
Age	0.475	−0.005	−0.019	0.009
Female sex	0.119	0.174	−0.045	0.393
Preoperative LVEF	0.547	0.433	−0.990	1.855
CPB time	0.022	0.014	0.002	0.026
Aortic cross-clamp time	0.642	−0.004	−0.019	0.012
Effect on log-transformed CK-MB levels at 6 h after surgery				
Modified Del Nido cardioplegia	0.495	0.094	−0.180	0.369
Age	0.764	−0.002	−0.017	0.012
Female sex	0.007	0.315	0.086	0.543
Preoperative LVEF	0.866	−0.126	−1.608	1.355
CPB time	0.003	0.019	0.007	0.031
Aortic cross-clamp time	0.125	−0.013	−0.029	0.004
Effect on log-transformed lactate levels at 6 h after surgery				
Modified Del Nido cardioplegia	0.118	0.206	−0.054	0.465
Age	0.619	0.003	−0.010	0.017
Female sex	0.843	−0.022	−0.238	0.195
Preoperative LVEF	0.597	−0.375	−1.778	1.029
CPB time	0.171	0.008	−0.004	0.020
Aortic cross-clamp time	0.310	−0.008	−0.023	0.008

## Data Availability

All important data are contained in the text of the manuscript. Raw data are available to the journal editor if requested.

## Abbreviations

ATP	adenosine triphosphate
AVR	aortic valve replacement
BMI	body mass index
BSA	body surface area
CK	creatine kinase
CK-MB	creatine kinase-myocardial band
CPB	cardiopulmonary bypass
Hs-TnI	high-sensitivity troponin
LVEF	left ventricular ejection fraction
MS	medial sternotomy
NYHA	New York Heart Association functional classification
PUMS	partial upper mini sternotomy
RAT	right anterior thoracotomy
